# Intraventricular dyssynchrony in light chain amyloidosis: a new mechanism of systolic dysfunction assessed by 3-dimensional echocardiography

**DOI:** 10.1186/1476-7120-6-40

**Published:** 2008-08-07

**Authors:** Raymond Q Migrino, Leanne Harmann, Timothy Woods, Megan Bright, Seth Truran, Parameswaran Hari

**Affiliations:** 1 Department of Medicine, Medical College of Wisconsin, Milwaukee, Wisconsin, USA

## Abstract

**Background:**

Light chain amyloidosis (AL) is a rare but often fatal disease due to intractable heart failure. Amyloid deposition leads to diastolic dysfunction and often preserved ejection fraction. We hypothesize that AL is associated with regional systolic dyssynchrony. The aim is to compare left ventricular (LV) regional synchrony in AL subjects versus healthy controls using 16-segment dyssynchrony index measured from 3-dimension-al (3D) echocardiography.

**Methods:**

Cardiac 3D echocardiography full volumes were acquired in 10 biopsy-proven AL subjects (60 ± 3 years, 5 females) and 10 healthy controls (52 ± 1 years, 5 females). The LV was subdivided into 16 segments and the time from end-diastole to the minimal systolic volume for each of the 16 segments was expressed as a percent of the cycle length. The standard deviations of these times provided a 16-segment dyssynchrony index (16-SD%). 16-SD% was compared between healthy and AL subjects.

**Results:**

Left ventricular ejection fraction was comparable (control vs. AL: 62.4 ± 0.6 vs. 58.6 ± 2.8%, p = NS). 16-SD% was significantly higher in AL versus healthy subjects (5.93 ± 4.4 vs. 1.67 ± 0.87%, p = 0.003). 16-SD% correlated with left ventricular mass index (R 0.45, p = 0.04) but not to left ventricular ejection fraction.

**Conclusion:**

Light chain amyloidosis is associated with left ventricular regional systolic dyssynchrony. Regional dyssynchrony may be an unrecognized mechanism of heart failure in AL subjects.

## Background

Light chain amyloidosis (AL) is a rare multiorgan disease with extracellular deposition of fibrillar amyloid proteins derived from immunoglobulin light chains [1,2]. Amyloid deposits in the heart, kidneys, liver and nervous system cause organ failure. There is poor prognosis with median survival of 4 months with heart failure [3,4]. It is associated with diastolic dysfunction but often preserved left ventricular (LV) ejection fraction, especially in the early stages [5-9]. Left ventricular dyssynchrony is common in heart failure patients and may contribute to its pathophysiology [10]. Intraventricular dyssynchrony reduces ventricular efficiency and cardiac performance [11] while cardiac resynchronization therapy improves symptoms and prolongs life [12,13]. Amyloid deposition can potentially alter regional cardiac mechanics. In a recent paper, Bellavia, et al. reported that patients with less advanced AL cardiac amyloidosis had increased segmental dyssynchrony compared to controls, but more advanced amyloidosis was associated with hypersynchronization using Doppler tissue velocity imaging [14]. Three dimensional (3D) assessment of regional dyssynchrony has potential advantage over 2-dimensional based tissue Doppler studies as the temporal relationships of all 16 segments can be related with ease. Recently, 3D echocardiography has been utilized to study the temporal pattern of the dispersion in segmental ventricular volumes during the cardiac cycle in the novel assessment of ventricular dyssynchrony [11,15] The dispersion (expressed as standard deviation) of the duration/timing from beginning of systole to the minimal systolic volume in the 16 different regions of the left ventricle (16-SD%, normalized to cycle length) has been shown to be a marker of dyssynchrony that was associated with ventricular dysfunction [11,15]. We hypothesize that AL subjects have left ventricular dyssynchrony compared to healthy controls. The aim of the study was to compare 16-SD% in AL subjects versus healthy controls.

## Methods

### Patient Population

Ten consecutive biopsy-proven AL subjects undergoing workup at 1 institution and 10 healthy controls underwent 3D echocardiography (60 ± 3 versus 52 ± 1 years, p = NS; 5 females in each group). The diagnosis was initially confirmed by biopsy for light chain amyloid in kidneys (n = 4), cardiac (n = 3), bone marrow (n = 2), gastrointestinal tract (1), fat pad, axillary mass (n = 1 each). Among AL subjects, 7 had cardiac involvement as defined by cardiac biopsy or subendocardial late gadolinium enhancement on routine magnetic resonance imaging [16,17]. Three AL subjects without cardiac biopsy or late gadolinium enhancement on MRI had thickened anteroseptum or increased left ventricular mass index on echocardiography.

The study was approved by the local Institutional Review Boards (IRB) and is in compliance with the Helsinki Declaration. All healthy controls gave informed consent. 9 AL subjects signed informed consent as part of a prospective observational study of biopsy-proven AL subjects. 1 AL subject who had tissue biopsy confirmation of the disease at post-mortem did not provide informed consent and waiver of consent authorization was obtained from the IRB.

### 3D Echocardiography Imaging and Analysis

In all subjects, cardiac 3D full-volume datasets were acquired from the apical window using either an IE33 or Sonos 7500 echocardiograph and X3-1 and X4-2 full matrix-array transducer (Philips Medical Systems, Bothell WA). The full volume data sets consisted of 4 real-time subvolumes acquired during 4 cardiac cycles that are subsequently combined to create a full 3D pyramidal data set. The data sets all had evaluable endocardial borders.

The 3D volume dataset were analyzed by software (QLAB version 4.2, 3DQ Avanced, Philips Medical Systems, Bothell WA) similar to previously published procedure [11]. In brief, 2-dimensional orthogonal planes representing the standard apical 4-chamber, apical 2-chamber and LV short axis were oriented to bisect the LV and incorporate the true LV apex. Five anatomic landmarks were set that included septal, lateral, anterior, inferior mitral annulus and the apical endocardium in both beginning and end of systole. The software then recreated a 3D model of the endocardial border at beginning and end of systole using automated border detection algorithm. Manual correction of endocardial border was done if necessary. The software then performed volumetric analysis creating a cast of the LV cavity throughout the cardiac cycle.

The LV was divided into 16 segments (excluding the apex) as per American Society of Echocardiography recommendations [18]. The volume of each segment was plotted as a function of time throughout the cardiac cycle, with time normalized to the cycle length and expressed as % R-R interval to account for differences in heart rate. The time from end of diastole (beginning of systole) and minimal systolic volume was quantified for each segment and the standard deviation of these times for the 16 segments (16-SD%) was calculated. The 16-SD% has been previously shown to be a reliable measure of dyssynchrony [11,15,19].

Two investigators (RQM and LH) trained in 3D volume analyses independently measured the 3D dataset blinded to disease condition and measured the subjects in random order. The first investigator repeated the measurement greater than a week after the first measurement in random order and still blinded to subject condition.

Left ventricular mass index (LVMI) was calculated by Devereux's formula using the diastolic left ventricular internal diameter, anteroseptal thickness and inferolateral thickness [20]. Left atrial volume index was calculated using area-length method as per American Society of Echocardiography standards [21]. In AL subjects, the lateral mitral annular velocity (E'), mitral inflow velocity (E) and ratio (E/E') were obtained using standard pulsed spectral Doppler echocardiography [22].

### Data Analyses and Statistics

Data are expressed as mean ± standard deviation. Continuous variables were compared by unpaired Student's t-test for normally distributed data -or Mann-Whitney rank-sum test for non-normally distributed data. Correlation analysis was performed using Pearson's correlation. Intraobserver and interobserver agreement was assessed using intraclass correlation coefficient (ICC) analyses and method of differences by Bland-Altman. For the ICC, a two-way mixed model absolute agreement type was used [23]. In this analysis, ICC values less than 0.4 indicate poor reproducibility, values between 0.4 to 0.75 indicate good reproducibility and greater than 0.75 shows excellent reproducibility [23,24]. Limits of agreement were assessed by plotting the differences in the measurement of 16-SD% against the average values of the measurement as described by Bland-Altman [25,26]. Analyses were performed using SPSS 16.0.1 (SPSS Inc. Chicago IL). A two-sided p-value less than 0.05 was used to denote statistical significance.

## Results

Eight AL patients had New York Heart Association functional classification I, with 1 each presenting in Class III and IV heart failure. Left ventricular ejection fraction was 62.4 ± 0.6% in control subjects and 58.6 ± 2.8% for AL (p = NS) (Table 1). AL subjects had thicker anteroseptum, increased left atrial volume index and tendency towards increased left ventricular mass index. The lateral mitral annular velocity (E') was 15.4 ± 9.2 cm/s and ratio of mitral inflow velocity to E' (E/E') was 7.2 ± 3.3 in AL subjects. Although E and E/E' data were not available in control subjects, the mean values of E' and E/E' in AL subjects in addition to increased left atrial volume index are consistent with diastolic dysfunction and increased left ventricular filling pressures based on prior validation studies [27-30]. Based on conventional evaluation of degree of diastolic dysfunction using mitral inflow and mitral annular velocity [31], 1 AL patient had normal diastolic function, 3 had mild (impaired relaxation pattern), 4 had moderate (pseudonormalization) and 2 had severe (restrictive) diastolic dysfunction.

**Table 1:** Echo parameters in AL and healthy subjects.

	**Control**	**AL**	**p-value**
LVEF (%)	62.4 ± 0.6	58.6 ± 2.8	NS
Anteroseptal thickness (cm)	0.97 ± 0.1	1.62 ± 0.5	**0.01**
LVMI (g/m2)	125.0 ± 48	179.3 ± 79	0.08
LAVI (mL/m2)	25.7 ± 4.8	39.6 ± 16.9	**0.009**
Cycle length (ms)	1060 ± 166	752 ± 211	**0.002**
16-SD% (%)	1.67 ± 0.87	5.93 ± 4.4	**0.003**

**Figure 1:**
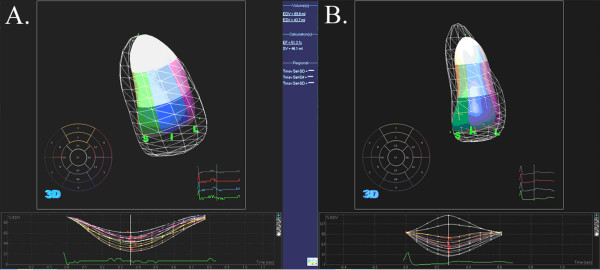
**3D echocardiographic analysis of segmental left ventricular volume change**. **A and B**. A represents data from a healthy subject and B represents data from AL subject. Top panel shows 16 segments analyzed (excludes apex) with solid cast representing endsystolic volume and surrounding grid cast representing beginning systole (or end-diastole) volume. The bottom panel shows the regional volume expressed in proportion to end-diastolic volume with time during the cardiac cycle. In these 2 examples, the dispersion of timing of endsystole is narrower in A compared to B.

**Figure 2:**
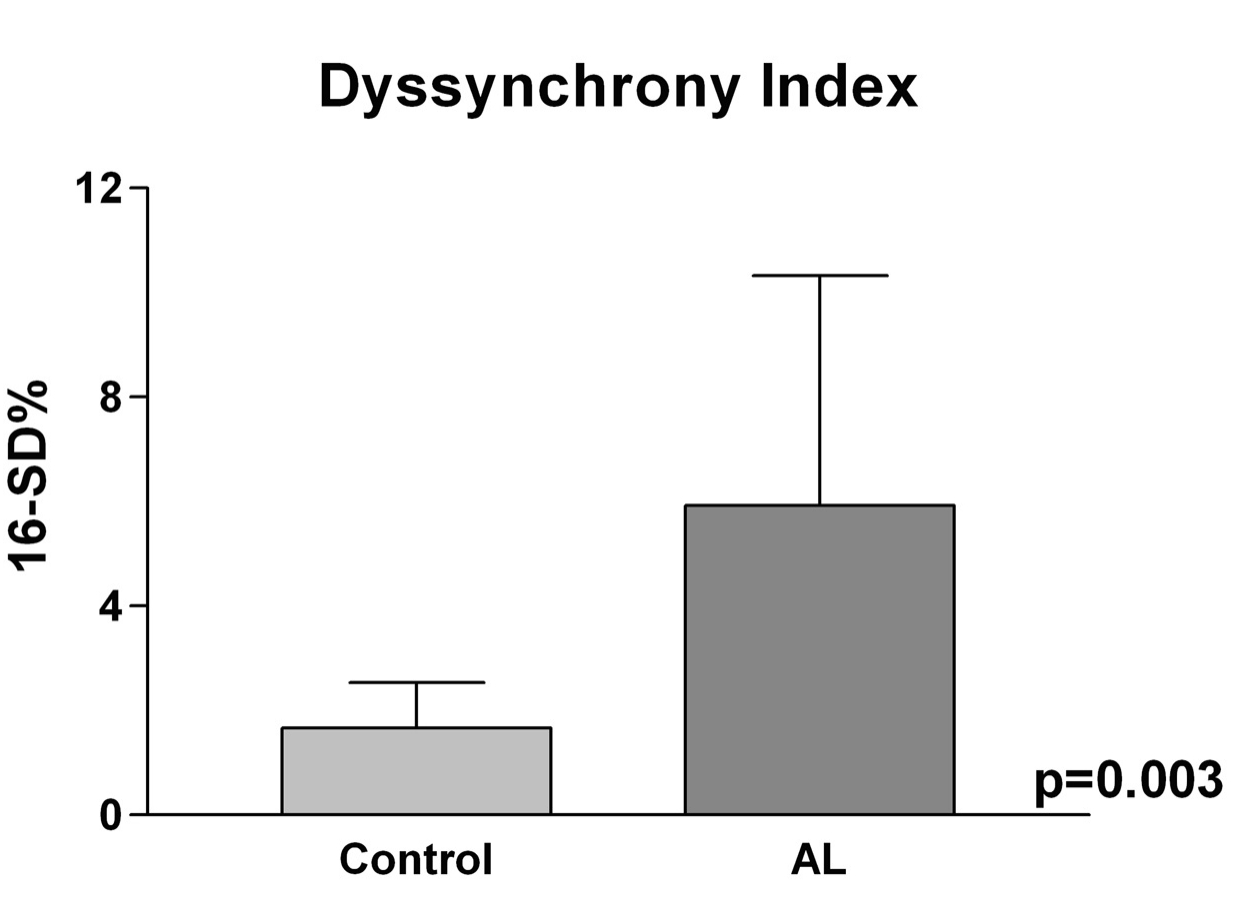
**16-SD%**. 16-SD% as a measure of regional ventricular dyssynchrony was significantly higher in AL compared to control subjects.

There was higher 16-SD% in AL subjects compared to controls (Table 1, Figures 1, 2, see Additional files 1, 2). There was shorter cycle length in AL subjects compared to controls. There was no correlation between cycle length and 16-SD% (R = -0.2, p = NS). 16-SD% was weakly correlated with left ventricular mass index (R = 0.45, p = 0.04). There was no correlation between 16-SD% and left ventricular ejection fraction (R = -0.3, p = 0.14).

**Figure 3:**
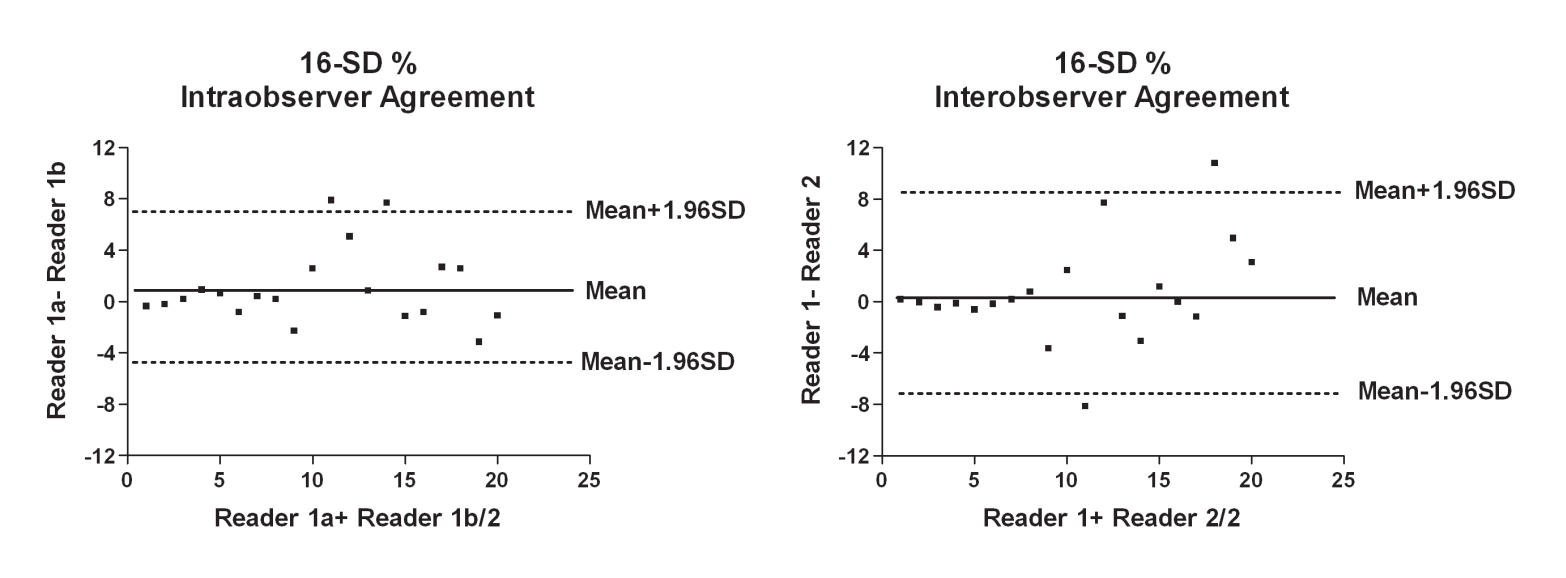
**Bland-Altman plot for intraobserver and interobserver limits of agreement for 16-SD%**. Differences in measurement are plotted against average of two measurements for intraobserver (left) and interobserver (right). There is overall good agreement especially at lower values of 16-SD%.

Intraclass correlation coefficient was 0.625 (p = 0.001) for intraobserver and 0.606 (p = 0.002) for interobserver differences in 16-SD%. The ICC together with the Bland-Altman analysis (Figure 3) show good reproducibility of 16-SD% measurements. The Bland-Altman plot further shows better agreement in the measurement at lower 16-SD% values.

## Discussion

Light chain amyloidosis is an often fatal disease with about 2500 new cases per year in the United States [3,32-35]. Patients with cardiac involvement have the worst outcomes in the presence of heart failure [3,36]. Amyloid deposition in the heart leads to ventricular thickening and impaired relaxation, classically presenting as diastolic dysfunction often with preserved systolic function as measured by left ventricular ejection fraction [5-8]. Indeed, left ventricular ejection fraction was found not to be an independent risk factor for mortality in this disease [4]. The elevated mitral annular velocity E' and ratio of transmitral inflow velocity to mitral annular velocity E/E' together with increased left atrial size suggest diastolic dysfunction [27] that is more prominent than conventional measures of systolic function (mean left ventricular ejection fraction 58.6 ± 2.8%) in our cohort of AL subjects.

Heart failure is an important cause of morbidity and mortality in AL amyloidosis. There is increasing recognition that timing of regional ventricular contraction or ventricular synchrony is important in the development of cardiac dysfunction and heart failure. Both interventricular and intraventricular dyssynchrony reduce ventricular efficiency and performance [11]. Treatment of dyssynchrony with cardiac resynchronization therapy has been shown to improve survival, reduce heart failure symptoms and improve quality of life [12,13,37-39]. 3D echocardiography is a recent advance in the volumetric assessment of left ventricular function and software allows the quantification of the dispersion of timing to peak systole among the different ventricular regions, a measure of intraventricular dyssynchrony. Early studies demonstrate that dyssynchrony measured by the dispersion of systolic timing among the 16 segments (16-SD%) is associated with systolic dysfunction [11,15]. In addition to quantifying the temporal relationship among segments, 3D echocardiography also allows acquisition that does not require correct acquisition axis. In the current study, significant dyssynchrony was demonstrated in AL subjects. The presence of dyssynchrony in the setting of relatively preserved LV ejection fraction in the AL group suggests that systolic dysfunction may occur earlier than previously recognized and that amyloid deposit in the heart, although diffuse in distribution [40], still result in regional contractile dysfunction. The study points to the potential of 3D echocardiography to detect early changes of left ventricular systolic dysfunction in AL amyloidosis that might not otherwise be detected by conventional measures of systolic function. The degree of dyssynchrony is correlated with left ventricular mass index suggesting that 16-SD% abnormality may be an early indication of cardiac amyloid deposition.

The current finding needs to be related to the recent study of Bellavia, et al. [14] that utilized 2-dimensional tissue Doppler imaging to assess regional dyssynchrony. In this study involving AL amyloid patients with relatively preserved left ventricular systolic function, they found that patients with less advanced cardiac involvement (ventricular thickness < 1.1–1.2 cm and without restrictive filling pattern) had significantly higher standard deviation of timing of segmental longitudinal and radial strain signifying regional dyssynchrony compared to age and sex-matched controls. However, patients with advanced cardiac involvement (ventricular thickness > 1.1–1.2 cm or restrictive filling pattern) surprisingly had reduced regional dyssynchrony. Although the thickness of the ventricle is consistent with advanced classification in most of our subjects using Bellavia's thickness criteria, most of our subjects had mild-moderate dysfunction (70%) with only 20% having restrictive filling pattern. It is possible therefore, that our patients are more closely related in degree of disease to the no-advanced group in that study. Our sample size is too small to compare more advanced versus less advanced disease. However, 3D echocardiography is theoretically superior to 2-dimensional based techniques in assessing regional dyssynchrony if data is acquired in 1 cardiac cycle as it allows simultaneous measurement of systolic timing in all 16 segments versus non-simultaneous sequential determination in different views for 2-D based methods. Differences in systolic timing of segments taken at different time points may not only be due to regional dyssynchrony but may also be affected by beat to beat variation in cycle length and systolic timing. It remains to be seen whether hypersynchronization would be also observed in advanced cardiac amyloid patients using 3-dimensional echocardiography.

The 3D echocardiographic method of assessing ventricular dyssynchrony appears to have good, although not excellent, agreement when measured by the same or different observers based on intraclass correlation coefficient and Bland-Altman analysis. The reliability of this technique for use in AL amyloidosis subjects needs to be validated in a larger number of subjects. In future studies, comparing the presence and degree of dyssynchrony between amyloid subjects and other benign conditions presenting with ventricular thickening such as hypertension may be useful in determining whether this novel measure is clinically useful in the early diagnosis of AL cardiac amyloidosis.

## Conclusion

Intraventricular segmental dyssynchrony is demonstrated for the first time in light chain amyloidosis subjects compared to healthy controls with higher temporal pattern of dispersion of regional volume systolic change (16-SD%) on 3D echocardiography. Since left ventricular ejection fraction is relatively preserved in this cohort of AL subjects, regional intraventricular dyssynchrony by 3D echocardiography is a novel technique that may allow earlier detection of systolic dysfunction in this disease condition.

## Competing interests

The authors declare that they have no competing interests.

## Authors' contributions

RQM and PH contributed to conception and design of the study, data acquisition and analyses and drafting of the manuscript. LH, TW, MB and ST contributed to acquisition of data and manuscript review. All authors gave final approval of the manuscript submitted.

## Supplementary Material

Additional file 1 **Normal control subject**. Top panel shows motion of the left ventricle with regions color-coded by standard ASE segmentation. The mesh represents the end-diastolic position of the segments. Lower panel shows the volume of each segment as a percent of end-diastolic volume by time normalized to the cardiac cycle length. Note that the timing of peak systolic volume change occurs almost simultaneously in this normal control subject. Click here for file

Additional file 2 **AL patient**. A similar 3D echocardiographic acquisition as in Movie 1 but in an AL patient. Note that there is less synchronous regional motion. Bottom panel shows the dispersion of timing of peak systolic volume change in the different segments. Click here for file
